# When Seeing Is Better than Doing: Preschoolers’ Transfer of STEM Skills Using Touchscreen Games

**DOI:** 10.3389/fpsyg.2016.01377

**Published:** 2016-09-13

**Authors:** Elizabeth L. Schroeder, Heather L. Kirkorian

**Affiliations:** Human Development and Family Studies, University of Wisconsin – Madison, Madison, WIUSA

**Keywords:** STEM, digital games, touchscreens, preschoolers, transfer, learning

## Abstract

The purpose of this study was to examine the extent to which character familiarity and game interactivity moderate preschoolers’ learning and transfer from digital games. The games were based on a popular television show and designed to test skills related to STEM (science, technology, engineering, mathematics): numerical cognition (quantity of different sets) and knowledge of a biological concept (growth). Preschoolers (3.0–5.5 years, *N* = 44) were assigned to play one game and watch a recording of an experimenter playing the other game. Learning was assessed during pre-test and post-test using screenshots from the game. Transfer was assessed using modified screenshots (near) and real-life objects (far). Familiarity was assessed by asking children to identify the television characters and program. Findings indicate that the effectiveness of the games varied by age and condition: younger children learned from the quantity game, but only when they watched (rather than played) the game. They did not transfer this information in either condition. Conversely, older children learned from the growth game regardless of whether they played or watched. However, older children only demonstrated far transfer if they watched (rather than played) the growth game. Thus, preschoolers may benefit more by watching a video than by playing a game if the game is cognitively demanding, perhaps because making decisions while playing the game increases cognitive load. Character familiarity did not predict learning, perhaps because there was little overlap between the lessons presented in the television program and game. Findings from the current study highlight the need for more research into educational games and applications designed for preschoolers in order to establish whether, how, and for whom screen media can be educationally valuable.

## Introduction

Young children are increasingly exposed to educational games and applications for touchscreen devices. While many developers claim that their mobile applications hold educational value, researchers know little about whether, how, and for whom these new media can promote learning. This is particularly true for digital games targeting the areas of science, technology, engineering, and mathematics (STEM). Moreover, given the cognitive demands of using interactive media, it is unclear whether young children benefit more from actively playing or watching games, especially when they are not familiar with the game or its characters. The purpose of the current study was to examine the extent to which character familiarity and game interactivity moderate preschoolers’ learning and transfer from digital games.

### Why Focus on STEM Skills in Early Childhood?

The USA is lagging behind other countries in science and mathematics. According to a recent international study of the proportion of young people with college degrees, the USA has dropped to 17th in science and 25th in mathematics (U.S. Department of Education, 2012). Achievement gaps in STEM-related fields appear early and persist over time. For instance, Morgan et al. (2016) examined the age of onset, over-time dynamics, and underlying mechanisms of science achievement gaps in USA elementary and middle schools. The researchers found that science achievement gaps appear before first grade and continue through eighth grade. The authors suggest early intervention is key to reducing achievement gaps in science. Similarly, number skills during the preschool years predict mathematics competency years later (Duncan et al., 2007; Locuniak and Jordan, 2008; Geary et al., 2013).

Despite the importance of early science and math skills for later academic success, STEM skills are relatively understudied in preschool populations. Nonetheless, research demonstrates that preschool-age children are capable of understanding a range of science concepts such as scientific methods (e.g., observation, hypothesis testing), physics (e.g., gravity), and biology (e.g., life cycles; see Gelman and Brenneman, 2004, for review). For instance, Rosengren et al. (1991) demonstrated that preschoolers understand growth, a basic biological concept. In this study, preschoolers were shown pictures of juvenile and adult animals and then asked to identify which pictures represented each animal as an adult. The researchers found a high performance rate, suggesting that even 3-year-olds have an understanding that in order for growth to happen, a change must ensue (e.g., the animal grows from little to big). Perceptual features, such as the relative size of different creatures, may be particularly important cues that help young children generalize biological concepts (e.g., food chains; Gluckman et al., 2014).

Similarly, young children are able to demonstrate basic mathematical skills prior to formal education (see Clements and Sarama, 2009, for review). Discriminating between number sets is one example. This skill has been demonstrated in children as young as 6 months (e.g., Xu et al., 2005). As children’s math abilities grow, discriminating between sets develops into comparing and adding numerical sets without counting and resorting to guessing strategies (Barth et al., 2005). By the end of the preschool period, children are capable of comparing sets of objects based on numerosity. For instance, Barth et al. (2005) reported that 5-year old-children performed above chance (67%) when asked to compare sets of dots and identify which set was greater.

This growing body of literature indicates that young children are capable of demonstrating basic science and math skills, and that early STEM skills predict academic performance many years later. Therefore, it is vital to develop scalable, cost-effective interventions that prepare young children to be successful in science and math. We turn now to a discussion of educational media as potential tools for early intervention.

### Can Young Children Learn STEM Skills from Screen Media?

Decades of research have demonstrated that educational programs can teach young children a wide range of content and skills (see Fisch, 2004, for review). Longitudinal studies suggest that educational television exposure during the preschool years predicts readiness at school entry (Wright et al., 2001) and academic achievement at least as far as high school (Anderson et al., 2001). Moreover, the effectiveness of educational television appears to be far-reaching: Mares and Pan (2013) conducted a meta-analysis of research on the effectiveness of international co-productions of *Sesame Street* and found consistently positive results for cognitive outcomes (including quantity) and learning about the world (including environment and science).

Researchers have begun to evaluate educational games and mobile applications in light of the increase in children’s access to and use of interactive platforms such as tablet computers (Levine and Vaala, 2013). Some field experiments suggest that educational computer games can be effective at improving skills they are specifically designed to teach, such as pre-literacy and reading skills (e.g., Din and Calao, 2001; Segers and Verhoeven, 2005). Of particular relevance here, one previous study suggests that preschool-age children can learn math skills from digital games: Aladé et al. (2016) examined the effect of interactivity on preschoolers’ ability to learn about measurement (a basic math concept) from a touchscreen game. The authors found that preschool children can indeed learn a novel measurement skill from child-directed, educational media presented on a touchscreen device. Despite the apparent efficacy of digital games for teaching a range of skills, parents appear to be particularly skeptical about the value of screen media for teaching science skills in particular (Rideout, 2014), thus the current study was designed to examine children’s acquisition of both math and science skills.

Also of interest in the current study was whether children can transfer what they have learned to new problems. In order to transfer, children must develop a flexible mental representation of the educational content and recognize the connection between previously learned solutions and new problems (Fisch et al., 2005; Barr, 2013). In particular, children must recognize the deep-structure similarity (e.g., the two problems both require addition) and disregard differences in surface structure (e.g., one problem is about flowers and the other problem is about animals; Fisch et al., 2005). In the final section of this literature review, we consider factors that moderate preschoolers’ direct learning and transfer from screen media.

### What Conditions Lead to the Best Learning Outcomes for Educational Media?

Some young children clearly learn from some educational media some of the time. However, there is substantial variability in the effectiveness of educational media across different titles, individuals, and contexts. Here, we consider characteristics of the medium itself as well as characteristics of the viewer and testing situation that may moderate the effectiveness of educational media. While there are many factors that moderate the effectiveness of educational media, for current purposes we focus on three factors: the ease with which children can use the medium, children’s familiarity with media content, and the extent to which children have to generalize in the face of perceptual differences.

#### Media Characteristics: The Case of Interactivity

The extent to which young children learn from screen media depends in part on the extent to which media content (Fisch, 2004, 2013) and device interfaces (Strommen, 1993) place demands on working-memory resources. Young children may be better able to navigate a simple, intuitive touchscreen interface than a game controller or computer mouse (Revelle, 2013), enabling more individualized control. If preschool-age children are able to maintain control of the game, their attention, engagement, and interest will likely increase (Calvert et al., 2005). However, the extent to which young children benefit from interactive (versus non-interactive) media is unclear.

Interactive media has been defined as when a program’s output is determined by the user’s input (Investopedia, 2010). As in previous research (Aladé et al., 2016; Choi and Kirkorian, 2016; Kirkorian et al., 2016), we define media as an interactive medium as one in which the child touches the screen to play a game themselves rather than watching pre-recorded video (e.g., of the experimenter playing a game). Interactive media provide contingency and feedback, encouraging a more scaffolded learning experience (Revelle, 2013; Hirsh-Pasek et al., 2015). This control allows children to go at their own pace (Hirsh-Pasek et al., 2015). Further, the feedback from interactivity is immediate and tells the player whether their choices were correct or not, allowing the player to monitor progress and connect to the game (Gee, 2005; Hirsh-Pasek et al., 2015). Thus, it may be unsurprising that older preschool-age children appear to learn specific problem-solving strategies from touchscreen games. For instance, Huber et al. (2016) assessed problem solving among 4- to 6-year-old children using the Tower-of-Hanoi task. Children played either a real-life version of the game (disks on a peg board) or a digital version of the game on a touchscreen tablet. The authors reported the same rate of learning for those who played with real objects and with the digital game (Huber et al., 2016).

Despite the potential efficacy of digital games for learning, some titles may be more effective than others. Research with digital games is limited, but research with electronic books suggests that while some interactive features draw the reader’s attention to the story and produce better learning outcomes, titles with too many of these features can draw attention away from the story and hinder learning for preschoolers (see Bus et al., 2015, for review). Moreover, the specific conditions that lead to learning vary with age among younger preschoolers. For instance, 2-year-olds viewed videos on a touchscreen tablet in order to learn words (Kirkorian et al., 2016) or find hidden objects (Choi and Kirkorian, 2016). Some children interacted with an application that was specifically designed to guide attention to important information on the screen (e.g., asking children to touch the location of an object that was being labeled), while other children interacted with a more open-ended application that allowed more flexibility in how they viewed videos (e.g., letting them touch anywhere on the screen to continue). A third group of children watched non-interactive videos. Results indicated that younger (but not older) 2-year-olds learned from applications that guided attention, but not from applications that were more flexible or from non-interactive video. Similarly, Aladé et al. (2016) reported that children between 3 and 5 years of age were better able to transfer a measurement strategy from screen media to perceptually different stimuli when they watched a digital game than when they actively played the game. Thus, the potential benefits of interactive media may only be realized when the cognitive demands of playing the game do not exceed the child’s ability to both play the game and process the content.

#### Individual Characteristics: The Case of Familiarity

Fisch (2013) theorized that certain viewer characteristics, including prior knowledge and high working-memory capacity, help children learn from educational media. One characteristic, character familiarity, is of interest for the current study. Being familiar with a character includes identifying a character by name (Calvert, 2002; Lauricella et al., 2011). According to Fisch’s (2013) model, if a viewer is familiar with a character, they do not have to use working-memory resources to learn about the character and instead can focus on the content to be learned. Lauricella et al. (2011) tested this with 21-month-old toddlers using a seriation task. Toddlers watched either a familiar or unfamiliar puppet place cups in order from smallest to biggest and then nest smaller cups inside larger ones. Children were then given an opportunity to play with the real cups, and researchers scored their imitation based on nesting smaller cups inside of larger ones. Only those who watched the familiar puppet outperformed those in a baseline condition who did not see either video. Others have reported similar findings (e.g., Howard Gola et al., 2013).

Familiarity can also include experience with a particular title. If children are familiar with a particular program, they understand the format of the show (e.g., prompts inviting the audience to respond to questions), which may further support comprehension (Crawley et al., 2002). In support of this hypothesis, Piotrowski (2014) reported that children 3–5 years of age learned more from *Dora the Explorer* (a preschool show) when they were familiar with the program. In particular, the children who were familiar with the show benefited from invitations to respond to the character’s questions. While familiarity with an “interactive” television show appears to moderate learning, research has yet to establish the extent to which familiarity moderates children’s learning from truly interactive media, such as digital games.

#### Transfer Demands: The Case of Perceptual Similarity

With the aid of familiarity, preschool-age children are capable of transferring information from educational media to a variety of problems. However, children may have particular difficulty when they have to generalize to problems that are perceptually different from those depicted in screen media (Fisch et al., 2005; Barr, 2013). For instance, Crawley et al. (1999) reported that 3- to 5-year-old children were able to generalize a problem-solving strategy from *Blue’s Clues* (a preschool program) after just one viewing when the test problem was similar to those seen in the show; however, they were only able to transfer to problems with different surface features when they viewed the same episode five times. Similarly, Aladé et al. (2016) found that preschoolers generalized a measurement strategy after either watching or playing a digital game when pictures in the test stimuli resembled those seen in the game (e.g., other animals); however, the children only generalized this strategy to less similar pictures (e.g., robot) when they watched (rather than played) the game. Thus, transfer in the face of perceptual dissimilarity appears to be a difficult task that may be hindered by more cognitively demanding media experiences.

### Overview of the Current Study

Screen media have the potential to teach STEM skills to young children. However, the exact conditions that produce the best learning outcomes appear to vary by viewer characteristics, such as age and familiarity with the characters and program. While some children benefit from interactive media, others may benefit equally (or more) from viewing non-interactive demonstrations, especially when transferring to perceptually different problems. Research that directly assesses the extent to which young children can learn and transfer STEM-related skills from digital media is lacking. It is imperative that researchers identify whether, how, and for whom screen media may be educational in order to inform caregivers, educators, and practitioners about effective learning experiences for young children.

The current study was designed to examine the extent to which familiarity and interactivity affect preschoolers’ learning from STEM games. Preschoolers (3–5 years) played one STEM game and watched a recording of an experimenter playing another STEM game. The experimenter assessed prior skill knowledge before children experienced each game. Direct learning and transfer were assessed after each game. In addition, the researcher assessed each child’s familiarity (with the characters and program) and receptive vocabulary.

In line with Fisch’s (2013) capacity model, we predicted that prior knowledge related to the educational content (i.e., pre-test scores) and familiarity with the characters and program featured in the game (i.e., ability to identify and name characters and television program) would reduce cognitive load during the games and therefore lead to greater direct learning and transfer. Given that prior research has mixed results regarding interactivity (Aladé et al., 2016; Choi and Kirkorian, 2016; Kirkorian et al., 2016), the effect of playing games compared to watching game-play was an open research question. If playing games supports learning (e.g., increasing engagement, scaffolding learning, allowing children to learn at their own pace), then we expected direct learning and transfer to be higher when children played (rather than watched) the game. On the other hand, if playing games disrupts learning (e.g., increasing cognitive load), then we expected children to learn more from the game they watched (rather than played).

## Materials and Methods

### Participants and Design

The study was carried out in accordance with the recommendations of the University of Wisconsin-Madison Education and Social/Behavioral Science Institutional Review Board with written informed consent from all participants’ guardians. Participants were 44 preschoolers (27 males) between 3 and 5.5 years of age (*M* = 4.2 years, *SD* = 0.8 years) recruited through local preschools and mailing lists. As described in Section “Results,” preliminary analyses indicated that the impact of the games varied by age. Thus, for the purpose of analysis, the sample was divided into younger (*n* = 22, *M* = 3.56 years, range = 3.04–4.29 years) and older groups (*n* = 22, *M* = 4.86 years, range = 4.39–5.41 years).

Of the 27 parents (61% of sample) who responded to the parent survey, 17 (63%) identified their child as White/Caucasian (non-Hispanic), four (15%) as Asian/Pacific Islander, one (0.4%) as Black/African American, and one (0.4%) as Hispanic; the remaining four (15%) identified their child as other/mixed race. Parent education averaged 19.07 years (*SD* = 2.44, range: 14–24). Data were collected from October 2014 to April 2015.

Children were randomly assigned to groups within a 2(condition: play versus watch) × 2(game: growth versus quantity) × 2(order: play first versus watch first) mixed design, with condition and game as repeated measures. Half of the children played the growth game and watched the quantity game (*n* = 22, *M* = 4.15 years), while the remaining children played the quantity game and watched the growth game (*n* = 22, *M* = 4.27 years). The order of conditions was counterbalanced with the constraint that about half of the children were randomly assigned to play first, while the other half watched first.

The children were also randomly assigned to one of two question sets that were identical in structure but varied in specific content (e.g., asked to identify which of three sets of items contained “3” versus “5”, asked to sort pictures of chickens versus penguins in order of increasing age). Preliminary analyses indicated that performance did not differ by question set, so analyses collapsed across this variable.

### Parent Survey

Parents were asked to complete an online survey including demographic information, media use, and child’s familiarity with the children’s television show on which the games were based (*Dinosaur Train*). In order to estimate overall media use, parents reported the number of minutes that their child used different types of media on the previous day. Categories included viewing non-interactive video content (television program, DVD) on a television, computer, streaming device, or mobile device; playing a game on a computer, video-game console, handheld gaming device, or mobile touchscreen device; and using a digital reading device (Nook, LeapFrog). In order to assess children’s familiarity with *Dinosaur Train*, parents were asked how familiar their child was with the show (very, a little, not at all) and how often their child watched the show (4–5 days per week, 1–2 days per week, 1–2 days per month, never/almost never).

### Stimuli and Apparatus

**FIGURE 1:**
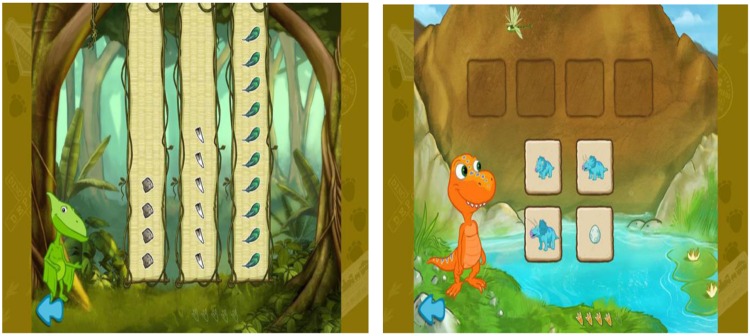
**Screenshot of the quantity game (*Don’s Collections*, **Left**) and growth game (*Life Cycles*, Right)**.

The touchscreen device used in this study was a *Samsung* Galaxy Tab 10.1. The children played and viewed professionally produced games based on the show *Dinosaur Train*. *Dinosaur Train* is a Public Broadcasting Station (PBS) television program that targets basic scientific thinking skills with a goal to teach about natural history, paleontology, and life sciences (PBS Kids, 2014). For this study, we used a low-cost mobile application based on *Dinosaur Train* entitled *Mesozoic Math Adventures*, targeting science and math skills in children 3–6 years of age (PBS Kids, 2014). The two games used in this study emphasized numerical cognition (e.g., quantity, set size) and the biological concept of growth (e.g., plants and animals get larger as they grow older). Screenshots from each game are depicted in **Figure 1**.

The quantity game, *Don’s Collections*, is designed to test children’s knowledge of collections, organization and presentation of data in a bar chart, and ability to compare different quantities of data (PBS Kids, 2014). Throughout the game, the character Don asks numerical comparison questions, such as: “Which one do I have the most of? Which one do I have the least of? Which one has more than this one? Which one do I have 5 of?” Children responded by touching the corresponding column. If the answer was incorrect, Don told the player that the answer was incorrect and suggested to try again. He then repeated the question and waited for the response. If the answer was correct, the game advanced to the next question. There were five collections with three questions each for a total of 15 questions.

The growth game, *Life Cycles*, is designed to test knowledge of life cycles and growth by putting organisms in order from youngest to oldest (PBS Kids, 2014). The growth game began with Buddy introducing his hypothesis (“Maybe little things grow into big things!”). The player was asked to put four tiles in order from youngest to oldest. Children moved the tiles to the spaces above by touching and dragging them to the corresponding location in the sequence. If a player moved a tile to an incorrect location, a red “X” appeared in the location and the tile automatically returned to its starting position. If the player was correct, a bell sound was played, signifying that the location was correct, and the tile locked into place. In other words, when a player was correct, they were no longer able to choose from the correct tiles, thus removing them as possible choices. In total, there were five trials with four tiles to complete on each trial.

**FIGURE 2:**
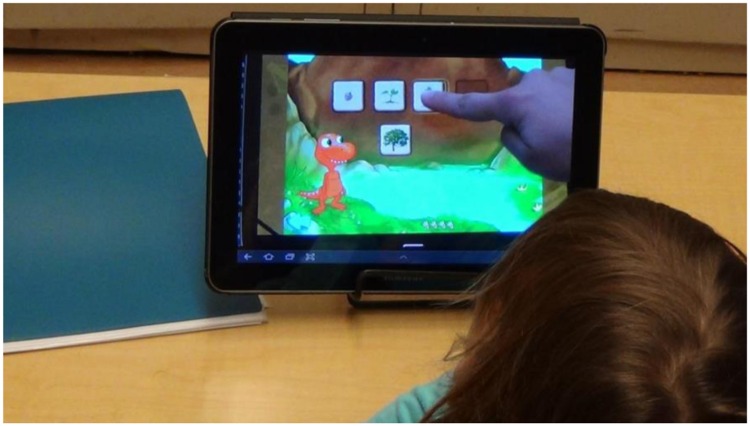
**Child watching a recording of an experimenter playing the growth game**.

Children were randomly assigned to play one of the games (as described above) and watch the other. In the watch condition, children viewed a video of an experimenter playing the game. In this condition, children could see a full-screen view of the game (as in the play condition) and the experimenter’s hand as she touched the screen to play the game (**Figure 2**). Thus in the watch condition, children only viewed correct game responses, and they could not control the pace of the game or alter its outcome.

### Procedure

**FIGURE 3:**
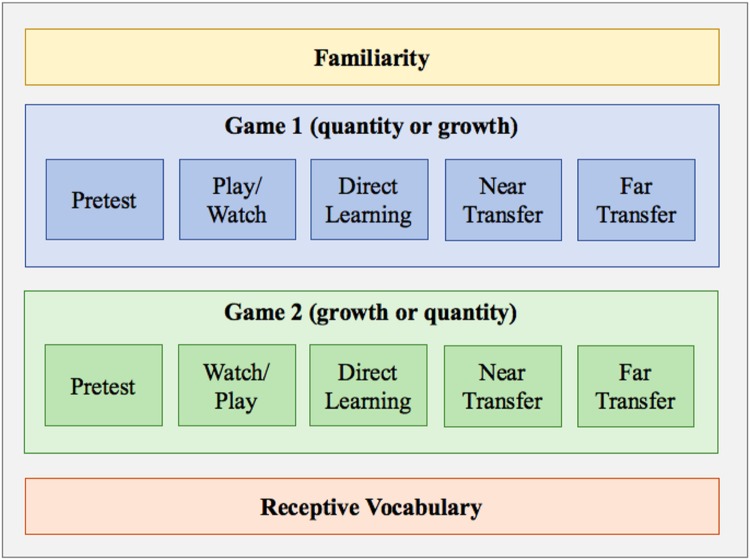
**Visual depiction of procedure; tasks were presented in order from top to bottom and left to right**.

Children were tested individually in an empty room at their preschool or in a laboratory on the university campus. **Figure 3** visually depicts the procedure, which lasted approximately 30 min. Each assessment is described in detail in the following sections. In brief, the general procedure was as follows: first, the child completed the familiarity assessment. Afterward, the child completed assessments for the first game (either watch or play, depending on the assigned condition). The order of the assessments for each game was: (1) pre-test to assess prior knowledge, (2) either play or watch the game, (3) post-test for direct learning, (4) post-test for near transfer, and (5) post-test for far transfer. After completing all post-test assessments for the first game, the child completed the assessments for the second game in the alternate condition (play or watch). After completing both games and learning assessments, the child completed a receptive vocabulary test.

### Assessments

#### Familiarity

In order to test their familiarity with the characters, the participants were shown a picture of the characters found in the games (Don and Buddy). The children were asked two questions of each character: (1)“Do you know who this character is?” and (2) “What is their name?” A final question asked whether they knew what program the characters were from, giving a total of five questions asked. The familiarity score was the sum of all questions answered correctly or in the affirmative (range: 0–5).

#### Prior Knowledge and Direct Learning

The purpose of this assessment was to determine whether children learned math and science skills from the games and could use that knowledge in the same context. This assessment was used at pre-test to assess prior knowledge and at post-test to assess direct learning. The format of the questions was the same during pre-test and post-test (e.g., asking which collection contained an exact number), but the specific content varied from one assessment to the next (e.g., asking which set contained 3 versus 5).

The experimenter showed the child screenshots taken directly from the games and asked questions using the same script as the hosts of each game. The only differences between this assessment and the game itself were that the children viewed printed screen shots for the assessments (rather than viewing on the touch screen) and responded to questions from the experimenter (rather than from the on-screen character). For example, in the quantity game, the child might be shown a printed version of the screenshot shown in **Figure 1** (left), and then be asked questions similar to those found in the game, such as “What does Don have the most of?,” “What does Don have 3 of?” Children were asked two questions for each of three screen shots, for a total of six questions at pre-test and another six questions at post-test.

Similarly, in the growth game, children were shown a printout of a screenshot such as that in **Figure 1** (right), with cutouts of the four pictures in the same location as they appeared in the game. Children were then asked to slide the cutouts onto the squares so that they appeared in order from youngest to oldest. Children were shown two screen shots at pre-test and another two screenshots at post-test.

#### Near Transfer

**FIGURE 4:**
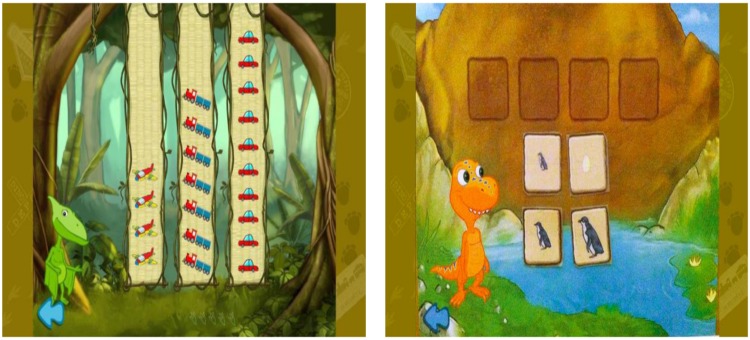
**Examples of images used in the near-transfer assessment for the quantity game **(Left)** and growth game (Right)**.

To succeed on the near transfer task, children were required to transfer what they learned in the game to a novel-but-similar scenario. The near-transfer task was identical to the direct-learning task except that images of contemporary objects and animals (e.g., trucks, chickens) replaced the thematically relevant ones that were found in the games (e.g., rocks, dinosaurs). These images were superimposed on the backgrounds used in the direct-learning test. For example, in the growth game, one item used is the lifecycle of a triceratops. In the near transfer task, the child was shown the lifecycle of a penguin. Both animals hatch from eggs, produce young that resemble the adult, and end with a larger adult animal. The questions were analogous to those asked in the direct-learning assessment. See **Figure 4** for examples of the near-transfer stimuli for the quantity and growth games.

#### Far Transfer

**FIGURE 5:**
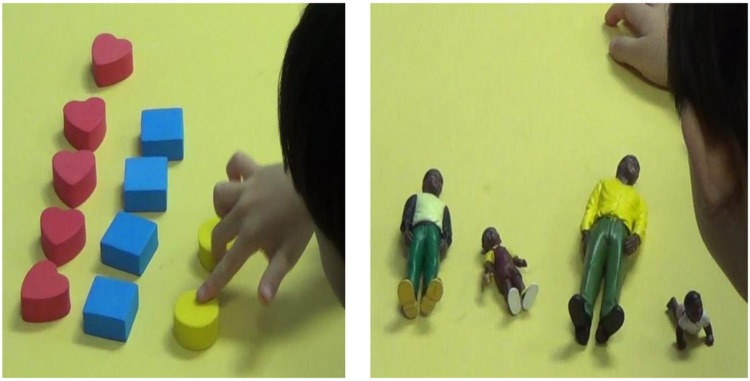
**Examples of stimuli used in the far-transfer assessment for the quantity game **(Left)** and growth game (Right)**.

To succeed on the far transfer task, children were required to transfer what they learned in the game to a scenario that was unrelated to *Dinosaur Train* using three-dimensional objects. Thus, the surface features of the far-transfer tasks differed substantially from the games: following the quantity game, children were asked questions about sets of foam blocks; following the growth game, children were asked to put dolls (infant, young child, older child, adult) in order from youngest to oldest (**Figure 5**). Despite the differences in surface features, the questions asked during the far-transfer tasks were analogous to those used for direct learning and near transfer.

#### Receptive Vocabulary

Receptive vocabulary was assessed using the Receptive One-Word Picture Vocabulary Test – Fourth Edition (ROWPVT-4). The ROWPVT – 4 is an individually administered, norm-referenced assessment of an individual’s ability to match a spoken word with a picture of its referent (Brownell and Martin, 2011). The distribution of standard scores has a mean of 100 and standard deviation of 15. Children viewed full-color pictures of four objects and were asked to point to the picture that matched a word (e.g., “Flower. Which one is flower?”). Children were asked increasingly difficult words until they answered six out of eight incorrectly. A standardized score was determined using norms based on the child’s age and sex. The distribution of standard scores has a mean of 100 and standard deviation of 15.

### Coding

#### Learning and Transfer

During each session, the experimenter noted the child’s responses to questions for pre-test and post-test assessments. For the quantity game, the experimenter recorded whether children selected the correct column (out of three) in response to each question. The dependent variable was the proportion of questions answered correctly during the pre-test and each of the three post-tests (direct learning, near transfer, far transfer). For the growth game, the experimenter initially recorded the order in which children placed the tiles or objects when asked to sort from youngest to oldest. The dependent variable was the proportion of tiles or objects that were placed in the correct location during the pre-test and each of the three post-tests.

#### Errors during Game Play

We recorded videos of experimental sessions for approximately 55% of the sample. For these children, videos were subsequently coded for the number of errors that children made while playing either the quantity game (*n* = 12) or the growth game (*n* = 12). For instance, in the quantity game, an error was scored if children selected an incorrect column (e.g., the column with the greatest number objects when asked for the column with the least number of objects); in the growth game, an error was scored if children dragged a tile to an incorrect location (e.g., tried to place the picture of the oldest animal in the spot for the youngest animal). The dependent variable was the proportion of all possible errors that were committed by children. For both games, the total possible errors across all questions equaled 30.

## Results

### Descriptive Statistics

Children’s mean vocabulary score was 111 (*SD* = 12, range = 83–137). When asked how much time their child spent using screen media on the previous day, parents reported an average of 33 min watching television (*SD* = 55, range = 0–240) and 6 min playing games on a touchscreen device (*SD* = 11, range = 0–30). When asked how familiar their child was with the program *Dinosaur Train*, parents reported “not at all” (23%), “a little” (38.5%), and “very” (38.5). When asked how often their child watched the program, parents reported “never or almost never” (38.5%), “infrequently (about 1–2 days per month)” (27%), and “some (about 1–2 days per week)” (34.5%). None of the individual difference measures (e.g., vocabulary, parent education, media use) were associated with any of the outcome measures of interest, so they are not considered further.

### Correlations between Familiarity, Prior Knowledge, and Learning

We hypothesized that children’s familiarity with the characters and program would predict direct learning and transfer from the games. Familiarity as measured in the lab (based on children’s recognition of and ability to name the characters and program featured in the game) was marginally correlated with parent-reported familiarity with the show (*r* = 0.35, *p* = 0.091) and frequency viewing the show (*r* = 0.35, *p* = 0.084). However, familiarity with the characters and show was not correlated with performance on any of the learning assessments for either game. **Table 1** depicts correlations between scores on the pre-test and three post-tests in the watch condition (above the diagonal) and play condition (below the diagonal).

**Table 1:** Partial correlations (controlling for age) between familiarity and learning assessments.

	1	2	3	4	5
(1) Familiarity	-	0.04	-0.25	0.01	0.04
(2) Pre-test	0.05	-	0.47**	0.35*	0.39*
(3) Direct learning	0.08	0.12	-	0.44**	0.51***
(4) Near transfer	0.03	0.21	0.70***	-	0.24
(5) Far transfer	-0.07	0.09	0.34*	0.51***	-

Familiarity was based on recognition and identification of the characters and television program. Learning assessments included the proportion of questions answered correctly during pre-test (prior knowledge), direct learning, near transfer, and far transfer. Numbers above the diagonal are for the *watch* condition, while those below the diagonal are for the *play* condition. *df* = 41. *p < 0.05, **p < 0.01, ***p < 0.001.

We further hypothesized that prior knowledge (measured at pre-test) would be associated with greater learning and transfer (measured at post-test). As can be seen in **Table 1**, pre-test scores were significantly correlated with post-tests in the watch condition only. In the play condition, pre-test scores were not associated with direct learning or transfer. However, direct learning from the game was associated with near and far transfer in both conditions.

### Learning from Watching versus Playing Games

Of particular interest in the current study was the impact of interactivity on learning and transfer. The omnibus analysis was a 2(age group: younger, older) × 2(game played: quantity, growth) × 2(condition: watch, play) × 4(test: pre-test, direct learning, near transfer, far transfer) mixed analysis of variance (ANOVA) with condition and test as repeated measures. The dependent variable was the proportion of questions answered correctly on each of the four tests. This analysis revealed several significant interactions, including a four-way interaction between all factors, *F*(3,120) = 5.55, *p* = 0.001, ?^2^ = 0.12. Visual inspection of the data revealed that the pattern of results differed for younger and older children and for each game. In order to capitalize on the within-subjects design of the study, and to address key hypotheses regarding direct learning and transfer (compared to prior knowledge assessed at pre-test), subordinate analyses entailed paired-samples *t*-test comparing each post-test assessment to pre-test for younger versus older children, for each game, and for each condition. The pattern of results was different for each game, so they are discussed separately.

#### Quantity Game

**FIGURE 6:**
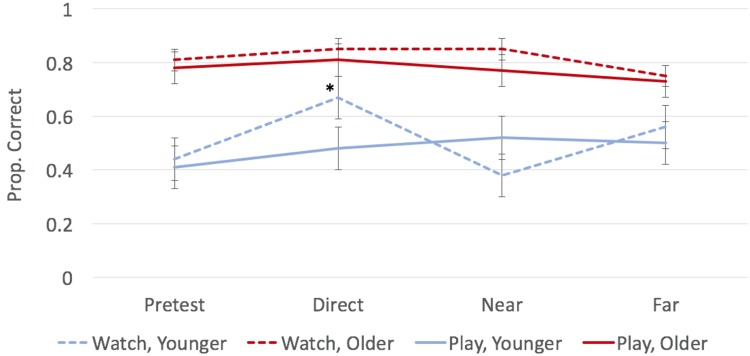
**Average proportion of quantity questions answered correctly during each test as a function of age and condition**. Bars represent ± one standard error. Points marked with an asterisk (*) indicate significant difference from pre-test at *p* < 0.05.

Quantity scores are plotted as a function of age and condition in **Figure 6**. It seems that the quantity game was too simple for the older children. Pre-test scores were already over 75%, thus post-test scores were (unsurprisingly) not significantly different from pre-test scores (all *p*s > 0.250).

Younger children were able to learn from the quantity game, but only when watching the game (not when playing it). Younger preschoolers who watched this game had higher scores on the direct post-test assessment than on pre-test, *t*(12) = 3.21, *p* = 0.008, *d* = 0.90. However, those who played this game did not do better on the direct assessment than on pre test, *p* > 0.250, *d* = 0.08. Even though younger children were able to learn from this game (in the watch condition only), this learning did not generalize to near and far transfer (*p*s > 0.250).

#### Growth Game

**FIGURE 7:**
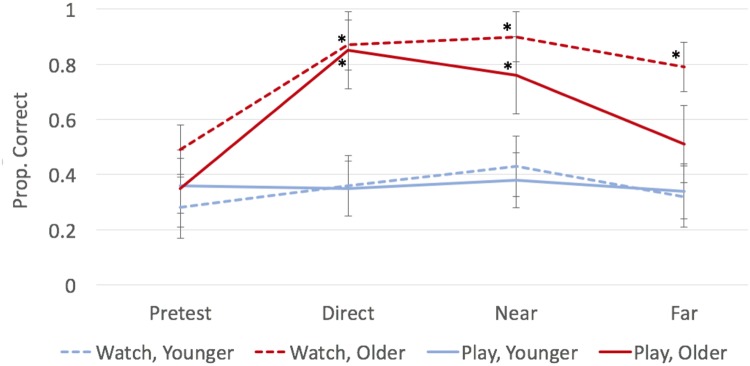
**Average proportion of growth questions answered correctly during each test as a function of age and condition.** Bars represent ± one standard error. Points marked with an asterisk (*) indicate significant difference from pre-test at *p* < 0.05.

Growth scores are plotted as a function of age and condition in **Figure 7**. Whereas, the quantity game appeared to be too simple for older children, the growth game appeared to be too difficult for younger children. There was evidence of a floor effect for this game, insofar as no post-test scores exceeded the relatively low pre test scores for younger children (all *p*s > 0.10).

Older children, on the other hand, did seem able to learn from the growth game. Direct learning scores were significantly greater than pre test scores in both the watch and play conditions, *t*(12) = 3.67, *p* = 0.003, *d* = 1.07, and *t*(8) = 3.24, *p* = 0.012, *d* = 1.15, respectively. Moreover, this learning generalized to the near-transfer test, which exceeded pre test in both the watch and play conditions, *t*(12) = 3.73, *p* = 0.003, *d* = 1.19, and *t*(8) = 2.34, *p* = 0.047, *d* = 0.79, respectively. However, learning in the growth game generalized to the far-transfer test only in the watch condition, *t*(12) = 2.36, *p* = 0.036, *d* = 0.66. The older children did not do better on far transfer than on pre test when they played (rather than watched) this game (*p* > 0.250, *d* < 0.25).

### Correlations between Game Errors and Subsequent Learning

Although children saw an errorless execution of the game in the watch condition, they were free to make errors in the play condition. We scored the number of errors made by children when playing one of the two games. Of particular interest was the extent to which the number of errors during the game was associated with tests of direct learning and transfer after the game. We calculated partial correlations between frequency of errors and post-test scores, controlling for age and pre test score. The number of game errors was negatively correlated with post-test measures of direct learning and near transfer, *r*(20) = -0.56, *p* = 0.007, and *r*(20) = -0.70, *p* < 0.001, respectively. In other words, children who made fewer errors while playing the game also performed better on tests of direct learning and near transfer, regardless of age and prior knowledge at pretest. However, the correlation between game errors and far transfer was not significant (*p* > 0.250).

## Discussion

Decades of research has demonstrated that preschool-aged children can learn a wide range of knowledge and skills from educational media (Fisch, 2004; Anderson and Kirkorian, 2015). However, research on interactive media has not kept pace with young children’s access to and use of digital games and mobile applications that purport educational value. Given the importance of early STEM skills for later academic success, it is crucial that researchers establish whether, how, and for whom educational media may foster early learning in these domains. The current project was designed to examine the impact of interactivity on young children’s direct learning and transfer from games that emphasize math and science skills, and the extent to which child characteristics (familiarity, prior knowledge) are associated with learning from these games.

### Associations between Familiarity, Prior Knowledge, and Learning

We predicted that familiarity would reduce cognitive load, and therefore be correlated with greater learning and transfer from the game. Contrary to this prediction, we found character familiarity was not correlated with any learning outcomes. In previous research, familiarity with both characters (Lauricella et al., 2011) and programs (Piotrowski, 2014) has been found to increase toddlers’ learning from video. However, the relation between familiarity and learning is not straightforward. For instance, Kirkorian et al. (2012) found that toddlers were not more likely to imitate a familiar (versus unfamiliar) character, despite attending more to the demonstration performed by a familiar character (as measured by eye movements). Thus, character familiarity does not always lead to increased learning.

In the current study, we tested children’s familiarity with characters from a popular television show (*Dinosaur Train*). However, we assessed learning from games based on the show, rather than the show itself. The game emphasized lessons that are not central to the television show (e.g., numerical cognition), and the format was substantially different from that in the television show (e.g., characters in the game spoke directly to the audience, asked questions, and provided feedback). Thus there may be limits to the benefit of character familiarity, depending on similarities between different learning contexts (e.g., show versus game).

We also hypothesized that prior knowledge (i.e., pre-test scores) would lead to greater direct learning and transfer. Interestingly this hypothesis was supported only when children watched a game; prior knowledge was not associated with learning when children played a game. The reason for this difference is unclear. Perhaps the act of playing the game drew more attention to the game mechanics rather than the educational lesson. As a result, children may have invested more effort in remembering the gestures required to interact with the game (e.g., tap in the quantity game versus slide in the growth game) than remembering their prior conceptual knowledge that would help them to answer questions correctly.

### Impact of Interactivity on Direct Learning and Transfer

Prior research demonstrates that young children have difficulty transferring information from video, particularly when test problems differ substantially from examples provided in the video (i.e., far transfer; Crawley et al., 1999). Research has been mixed regarding whether interactivity during a game would enhance or impede subsequent learning from that game. While interactivity may support learning, the specific conditions that lead to the best learning outcomes appear to vary with age, at least among younger preschoolers (Choi and Kirkorian, 2016; Kirkorian et al., 2016). Moreover, Aladé et al. (2016) found that playing a digital game (as opposed to watching a recording of that game) may be particularly detrimental to transfer. Specifically, they found that 3- to 5-year-old children applied a measurement strategy to images that resembled those presented in a game, regardless of whether they played the game themselves or watched a recording of the game. However, they only applied the measurement strategy to images that differed from those presented in the game when they watched a recording of the game.

Findings from the current study replicate those of Aladé et al. (2016) using games that purport to teach skills related to numerical cognition (e.g., number, set size comparison) and biological concepts (e.g., growth, life cycles). Moreover, our findings extend prior research by demonstrating a developmental progression in the extent to which children learn and transfer from interactive and non-interactive experiences. Younger children were able to learn from one of the games, but only when they watched a recording of the game; children who actively played the game themselves did not demonstrate pretest–posttest gains. Moreover, learning in the watch condition did not extend to transfer, even when using backgrounds that were identical to those in the game (near transfer). Thus, younger preschoolers had difficulty generalizing information beyond the digital game, and they only did so when cognitive load was relatively low (i.e., direct learning in the watch-only condition).

Older children, on the other hand, demonstrated both direct learning and near transfer from one of the games, regardless of whether they played or watched that game. However, learning only generalized to far transfer with three-dimensional objects when children watched a recording of the game; performance on the far transfer task did not exceed performance at pre-test when children played the game themselves. As in the study by Aladé et al. (2016), it seems that interacting with the game prevented children in the current study from transferring to perceptually different problems.

Together our findings suggest that children may learn equally well when watching or playing a game when the task is well within the child’s abilities (e.g., direct learning among older preschoolers). However, watching a game may be more beneficial than playing a game when the task is at the upper limits of the child’s abilities (e.g., direct learning among younger children, far transfer among older children).

Any generalization of information from educational media to real-life scenarios requires that children form flexible representations that can be readily applied in a variety of contexts (Fisch et al., 2005; Barr, 2013). Perhaps the additional cognitive burden of interacting with a game prevents children from extracting the deep structure of problems, and instead leads them to “over-encode” the surface features (e.g., particular images in the games, gestures required to play the game). Indeed, this interpretation is consistent with Aladé et al.’s (2016) finding that preschoolers who played a game outperformed those who watched a recording of the game when the test involved images that were perceptually similar to those presented in the game. Thus, interactive features may support direct learning at the expense of transfer to perceptually dissimilar scenarios.

### Implications and Future Directions

Current findings suggest that young children can learn from digital games, but that transfer from these games may be particularly difficult. Children may benefit most from non-interactive media when task demands are high. However, it is important to note that these findings are based on a convenience sample of mostly White/Caucasian and highly educated families. Further research is needed to determine generalizability of these findings. Achievement gaps in math and science appear early and persist over time, thus it is critical for future research to explore the efficacy of both interactive and non-interactive educational media among a socioeconomically diverse sample of children.

Further, it is important to emphasize that children in this study watched a flawless execution of one game but were free to make errors when playing the other game. Although some research suggests that incorrect examples help school-age children learn (Durkin and Rittle-Johnson, 2012; Booth et al., 2013), other research suggests that this practice only benefits advanced school-age students (Heemsoth and Heinze, 2014). Those students with relatively low prior knowledge, who may be considered more similar to preschool-age children, learned more from correct examples (Heemsoth and Heinze, 2014). Therefore, children in the current study may have learned less from playing (versus watching) games because they had conflicting memory of correct and incorrect responses to questions. This interpretation is supported by our own finding that children who made more errors when playing a game had lower scores on tests of direct learning and near transfer. However, the frequency of errors did not predict performance on far transfer assessments, perhaps because far-transfer scores were generally lower (and therefore less variable) than those for direct learning or near transfer. A follow-up study can more directly evaluate the hypothesis that correct examples support learning by comparing children in the current conditions to those who view a recording of an experimenter making errors while playing the game. Additionally, it may be that children take longer to master a concept when playing a game (particularly if they make many errors), but eventually develop greater mastery. Thus, future research should evaluate learning and transfer after repeated exposure to games, providing more time for children to learn and practice skills.

Finally, the current findings are limited to just one type of game. It is noteworthy that the games used in the current study did not start with a lesson to teach children about an underlying math or science skill. Thus children were only able to learn through trial and error, and the feedback provided by the characters in the game only indicated whether responses were correct or incorrect, rather than scaffolding children by explaining why answers were incorrect (Hirsh-Pasek et al., 2015). This would explain why playing the game in the current study did not lead to robust learning and transfer. Future research should examine particular features of digital games that lead to robust learning and flexible representations.

## Conclusion

Young children are using digital games at increasing rates, and many titles are advertised as educationally valuable. However, current findings demonstrate that learning and transfer cannot be assumed. The extent to which young children learn from screen media depends on a wide range of individual characteristics and media features, and young children may have particular difficulty generalizing information to new scenarios. Thus, it is critical to identify whether, how, and for whom educational media can be effective in order to maximize educational impact.

## Author Contributions

ES gathered information for the literature review, conducted the study, conducted analysis on the data, and wrote the manuscript as a part of her Master’s Thesis at University of Wisconsin – Madison. For publication purposes, ES worked on editing to fit the format of Frontiers. HK contributed to this manuscript by assisting with coding, data analysis, and editing as ES’ mentor at University of Wisconsin – Madison and co-author of this study.

## Conflict of Interest Statement

The authors declare that the research was conducted in the absence of any commercial or financial relationships that could be construed as a potential conflict of interest.
